# The efficacy and assessment value of the level of thyroglobulin wash-out after fine-needle aspiration cytodiagnosis in the evaluation of lymph node metastasis in papillary thyroid carcinoma

**DOI:** 10.1186/s12957-024-03430-5

**Published:** 2024-06-05

**Authors:** Jie Chen, Zongwu Lin, Bo Xu, Tianwen Lu, Xinghai Zhang

**Affiliations:** Thyroid and Breast Surgery, Department of General Surgery, Wanbei Coal and Power Group General Hospital affiliated With Bengbu Medical University, Su Zhou, Anhui Province China

## Abstract

**Objective:**

The purpose of this study was to evaluate the efficacy and clinical value of US, FNAC,FNA-Tg and FNAC + FNA-Tg, as well as the cutoff values of FNA-Tg to evaluate LN metastasis.

**Methods:**

We analyzed the diagnostic value of different US signs, the efficiency of US, FNAC, FNA-Tg and FNAC + FNA-Tg among the LN- and LN + groups, and the cutoff value of FNA-Tg to evaluate LN metastasis. We punctured LNs multiple times and measured the levels of FNA-Tg. Furthermore, the LNs were marked with immunohistochemical Tg and LCA to distinguish the presence of Tg in the para-cancerous tissue of the LNs.

**Results:**

The s-Tg and FNA-Tg of the LN + group were higher than those of the LN- group (*P* = 0.018, ≤ 0.001). The LN + group had more abnormal US signs than the LN- group. The cutoff value of FNA-Tg was 3.2 ng/mL. US had a high sensitivity (92.42), but the specificity was not satisfactory (55.1). FNA-Tg had a higher sensitivity (92.42 vs. 89.39), specificity (100 vs. 93.88), and accuracy (92.42 vs. 83.27) than FNAC. However, the sensitivity of FNAC + FNA-Tg increased further, while the specificity and accuracy decreased slightly. The presence of Tg in the normal lymphocytes adjacent to the cancer was confirmed.

**Conclusion:**

Ultrasonography provides a noninvasive, dynamic, multidimensional assessment of LNs. With a cutoff value of 3.2 ng/mL, FNA-Tg has higher accuracy and a lower false-negative rate than various single diagnoses. However, FNAC combined with FNA-Tg does not cause additional pain to patients and offers a higher diagnostic efficacy and clinical value.

**Supplementary Information:**

The online version contains supplementary material available at 10.1186/s12957-024-03430-5.

## Introduction

In the past 30 years, thyroid carcinoma has become the fastest-growing disease globally. As the largest proportion of differentiated thyroid cancer, the incidence of papillary thyroid carcinoma (PTC) can be more than 60%. Recurrence and persistent metastasis of cervical lymph nodes (LNs) occur in up to 30% of PTC patients [1]. “Small lesion with large metastasis” is not uncommon. Early detection and prompt and complete removal of metastasized LNs are factors affecting the range of interventions and the prognosis of PTC patients.

Ultrasonography (US) is the most important method to evaluate lymph node metastasis. However, its ability to distinguish benign from malignant lesions is insufficient. Jin Cho et al. showed the CT pooled sensitivity and specificity to evaluate LN metastasis to be 55 and 87%, respectively [2]. J Cho et al. reported the sensitivity and specificity of MRI to evaluate the metastasis of LNs to be 80 and 85%, respectively [3]. However, the variety of assessment methods makes it difficult to meet the needs of surgeons. Fine needle aspiration cytodiagnosis (FNAC) shows good clinical value in this context. This technique significantly improves the sensitivity and specificity of LN assessment. However, FNAC shows a 6–8% false-negative rate and a 5–10% inadequacy rate [4, 5]. The era of precision therapy requires improved assessment accuracy.

Since Pacini et al. first measured thyroglobulin in fine needle aspirate wash-out fluid (FNA-Tg) in 1992, this technique has been widely used and has shown promising results for improved diagnostic sensitivity of LN metastasis [6]. Although several studies have documented the usefulness of FNA-Tg, controversies remain about the optimal cutoff value. Furthermore, FNA-Tg lacks validation with large sample data and systematic evaluation.

This study evaluated the efficacy and clinical value of US, FNAC, FNA-Tg, and FNAC + FNA-Tg and the cutoff values of FNA-Tg. Our deep analysis assessed the evaluation of LN metastasis by FNA-Tg to provide a reference for clinical diagnosis.

## Materials and methods

### Patients and inclusion criteria

**Fig. 1:**
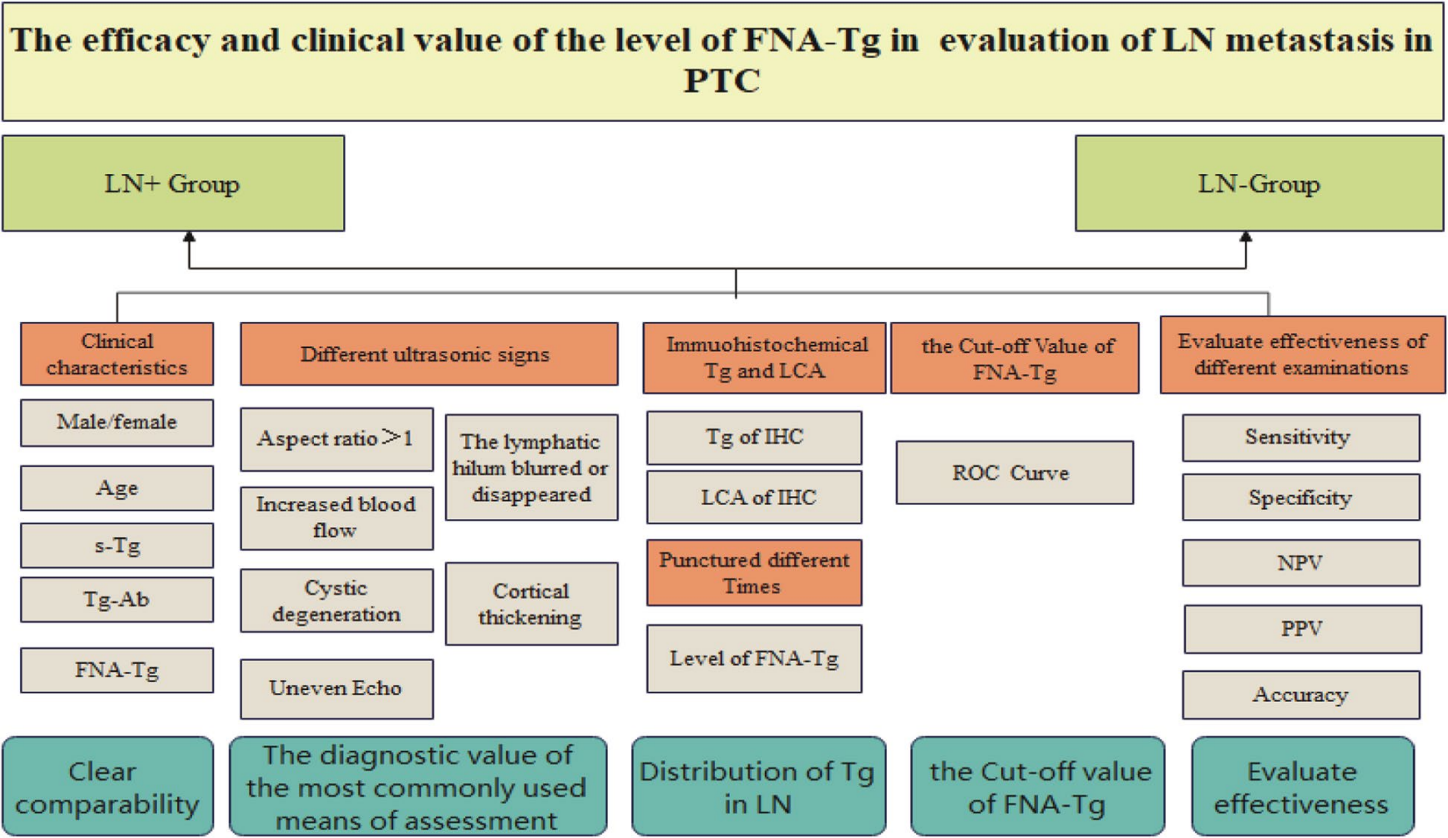
Technology roadmap

A total of 86 PTC patients with suspected LN metastasis were enrolled. Before being admitted, all patients were informed of the objectives, contents, and related details and signed the consent form. This project was approved by the ethics committee of Wanbei Coal and Electricity Group General Hospital (No. WBZY-LLWYH-2024–020).We examined all patients by US, FNAC, and FNA-Tg. Based on the results of repuncture or follow-up, patients were divided into two groups (lymph-node-negative (LN-) group and lymph-node-positive (LN +) group). We compared the diagnostic indicators of US, the test efficiency of the four methods of US, FNAC, FNA-Tg, and FNAC + FNA-Tg, and the cutoff values of FNA-Tg of the two groups to evaluate lymph node metastasis, as shown in Fig. 1.

The inclusion criteria were as follows: patients with (1) thyroid nodules considered malignant; (2) lymphadenopathy; (3) malignant LNs confirmed by pathological examination after biopsy; negative LNs confirmed by pathological examination, repuncture, or at least 1-year follow-up still without signs of transfer; (4) no other diseases and oral medication; and (5) denial of neck radiation.

### Ultrasonography

The color ultrasonic diagnostic instrument was a Samsung RS80A. An experienced sonographer assessed LNs in newly diagnosed lymphadenopathy patients with suspected thyroid cancer. We recorded different signs in the LN (ratio of the length to the diameter/absence of LN hilum/obfuscated cortex and medulla/cystic content/change in internal echo/increase in blood flow, and so on).

### FNAC

All patients underwent ultrasound-guided FNAC by the same experienced doctor and puncturing of LNs. For the puncture needle technique, a lengthened 5 ml syringe needle was chosen. Before each smear, the LNs were punctured 5–10 times in different directions and layers. Two punctures in the same position or level of the lymph node should be avoided as much as possible. The specimen was drawn and smeared on glass slides immediately. The slides were fixed with alcohol for seconds and reviewed by a professional pathologist.

### FNA-Tg, serum Tg, and Tg-Ab

Punctures were performed as described above. The needle was flushed with 1 mL of normal saline, and the eluent was analyzed for the Tg content. All patients underwent venipuncture to obtain blood samples to measure serum Tg and Tg-Ab. All tests were performed in the same hospital.

### Immunohistochemistry (IHC)

Antibodies used included Thyroglobulin antibody (Clone: IHC-M138(LBP1-TG), Leukocyte Common Antigen (LCA) antibody (Clone: IHC-M038(2B11&PD7/26)). All procedures sent to the pathology department by the unified production of professional and technical personnel, by more than 2 pathologists reading films.

### Index of observation and evaluation

① The eluent thyroglobulin concentration was checked by chemiluminescence. ② If the pathologist, who had more than three years of experience in cytology, did not obtain a clear reading, the case was reported to the superior doctor for consultation or repuncture. ③ The sensitivity, specificity, and accuracy of US, FNAC, FNA-Tg, and FNAC + FNA-Tg were evaluated and defined as follows: sensitivity = true positive cases/(true positive cases + false-negative cases)*100%. Specificity = true negative cases/(true negative cases + false-positive cases)*100%. ④ FNAC + FNA-Tg was judged to be positive when one of the two items was positive. It was judged to be negative when both items were negative.

### Statistical analysis

The data were analyzed with SPSS 21 statistical software. The mean ± standard deviation was used to express the measurable data. The chi-square test was used for the two diagnostic methods. We considered differences statistically significant at *P* < 0.05. We evaluated the FNA-Tg cutoff value using the ROC curve.

## Results

### s-Tg and FNA-Tg may be risk factors for positive lymph nodes

The study included 86 patients with 115 LNs (Table 1). The LN + group included 17 (36.17) patients over 55 years old, and the LN- group included 13 (33.33) patients over 55 years old. The LN + group included 16 (34.04) cases of male patients, and the LN- group included 13 (33.33) cases of male patients. There was no significant difference between the two groups, indicating that the basic information for the two groups was similar. There was no significant difference in Tg-Ab between the two groups. The s-Tg of the LN + group was higher than that of the LN- group (63.79 ± 95.87 vs. 33.88 ± 60.25, *P* = 0.018). The FNA-Tg of the LN + group was higher than that of the LN- group (159.57 ± 124.58 vs. 0.54 ± 0.61, *P* ≤ 0.001).

**Table 1:** Clinical characteristics of patients

Basic information	LN ( +)	LN (-)	Value
Total patients	47	39	
Total lymph nodes	66	49	
Age(≥ 55y / < 55y)	17(36.17)	13(33.33)	0.783
Male/female	16(34.04)	13(33.33)	0.945
s-Tg	63.79 ± 95.87	33.88 ± 60.25	0.018
Tg-Ab	182.11 ± 375.88	229.77 ± 537.70	0.089
FNA-Tg	159.57 ± 124.58	0.54 ± 0.61	< 0.001

*s-Tg* serum thyroglobulin, *Tg-Ab* thyroglobulin antibody, *FNA-Tg* thyroglobulin in the fine-needle aspirate fluid

Data are presented as the means ± SDs SD

### Detection of lymph nodes in patients with different ultrasonic signs

**Fig. 2:**
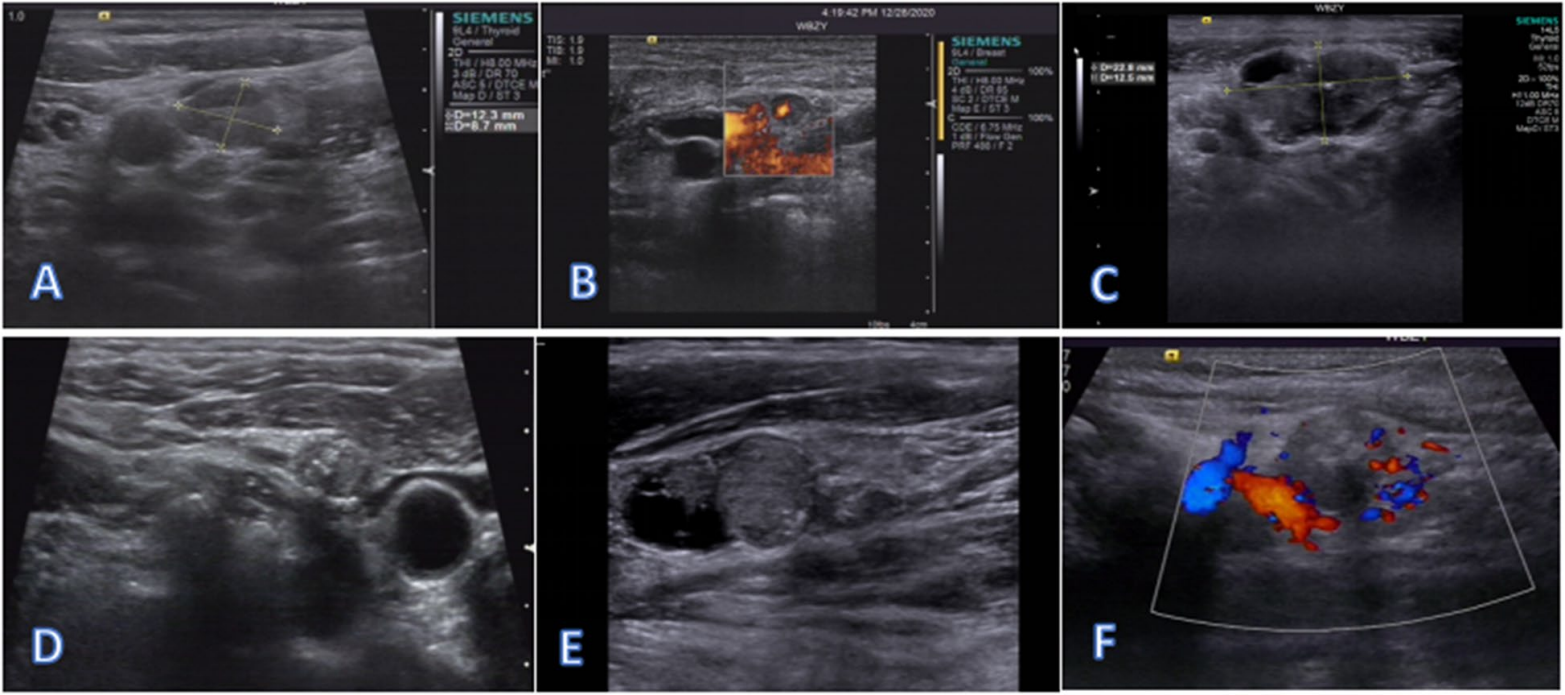
Different ultrasonic pictures of lymph node metastasis. **A** Cortical thickening. **B** aspect ratio > 1. **C** The lymphatic hilum was blurred or had disappeared due to cortical thickening. **D** Uneven Echo. **E** Cystic degeneration. **F** Increased blood flow signals

The metastasis of PTC to LNs changes ultrasonic features, including an aspect ratio > 1, increased blood flow signals, cystic degeneration, uneven echo, blurred or missing lymphatic hilum, cortical thickening, and others. These features are the main reference factors for ultrasound evaluation of metastasis. There was only 1 case of LN fusion fixation, and this patient had many LN metastases. Therefore, this study did not conduct statistical analysis (see the six pictures above). The number of cases with an aspect ratio > 1 in the LN + group was higher than that in the LN- group (46.97 vs. 24.49, *P* = 0.014), the number of cases with increased blood flow signals in the LN + group were higher than those in the LN- group (89.39 vs. 34.69, *P* < 0.001), the number of cases with uneven echo in the LN + group was higher than that in the LN- group (65.15 vs. 67.35, *P* = 0.806), the number with blurred or disappeared lymphatic hilum in the LN + group was higher than that in the LN- group (31.82 vs. 8.16, *P* = 0.002), and the number with cortical thickening in the LN + group was higher than that in the LN- group (63.64 vs. 16.33, *P* < 0.001). All 4 cases of LN cystic degeneration appeared in the lymph node metastasis group. However, the results were not statistically significant. A comprehensive analysis may show fewer cases of cystic lesions (Fig. 2).

### The lymph nodes were punctured different numbers of times. The paraffin blocks of PTC lymph node metastasis were selected and marked with immunohistochemistry TG and LCA

After 5 and 10 punctures of 4 lymph nodes, the levels of FNA-Tg were measured by the same method. FNA-Tg levels did not increase with the increased number of punctures.

**Fig. 3:**
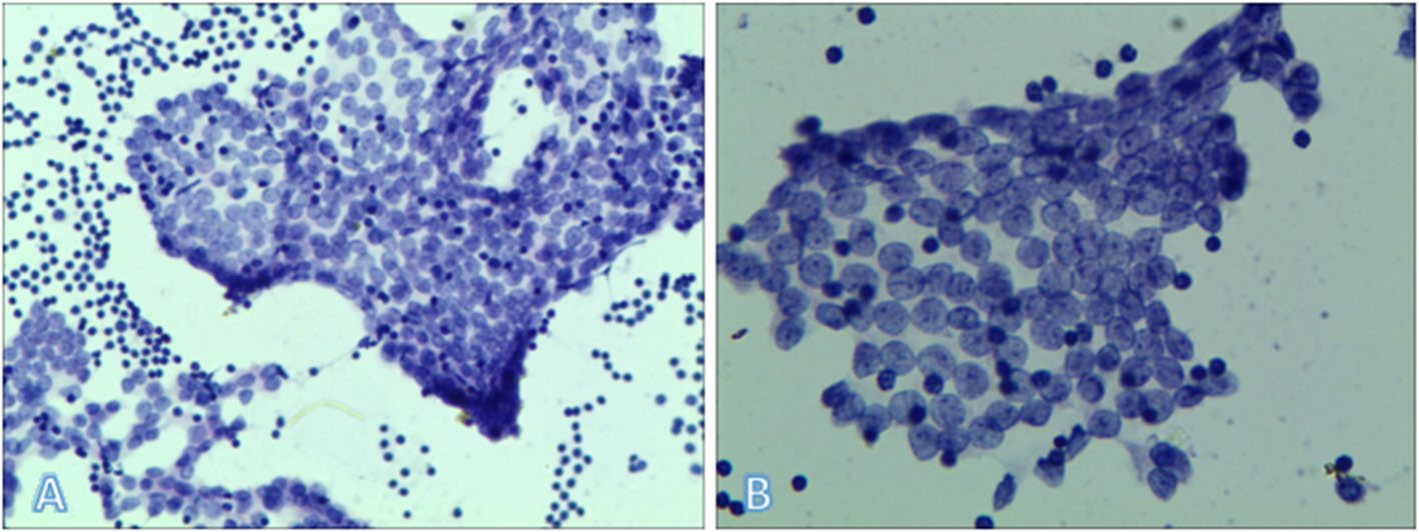
Fine-needle aspiration cytology of the lymph nodes: papillary thyroid carcinoma cells

**Fig. 4:**
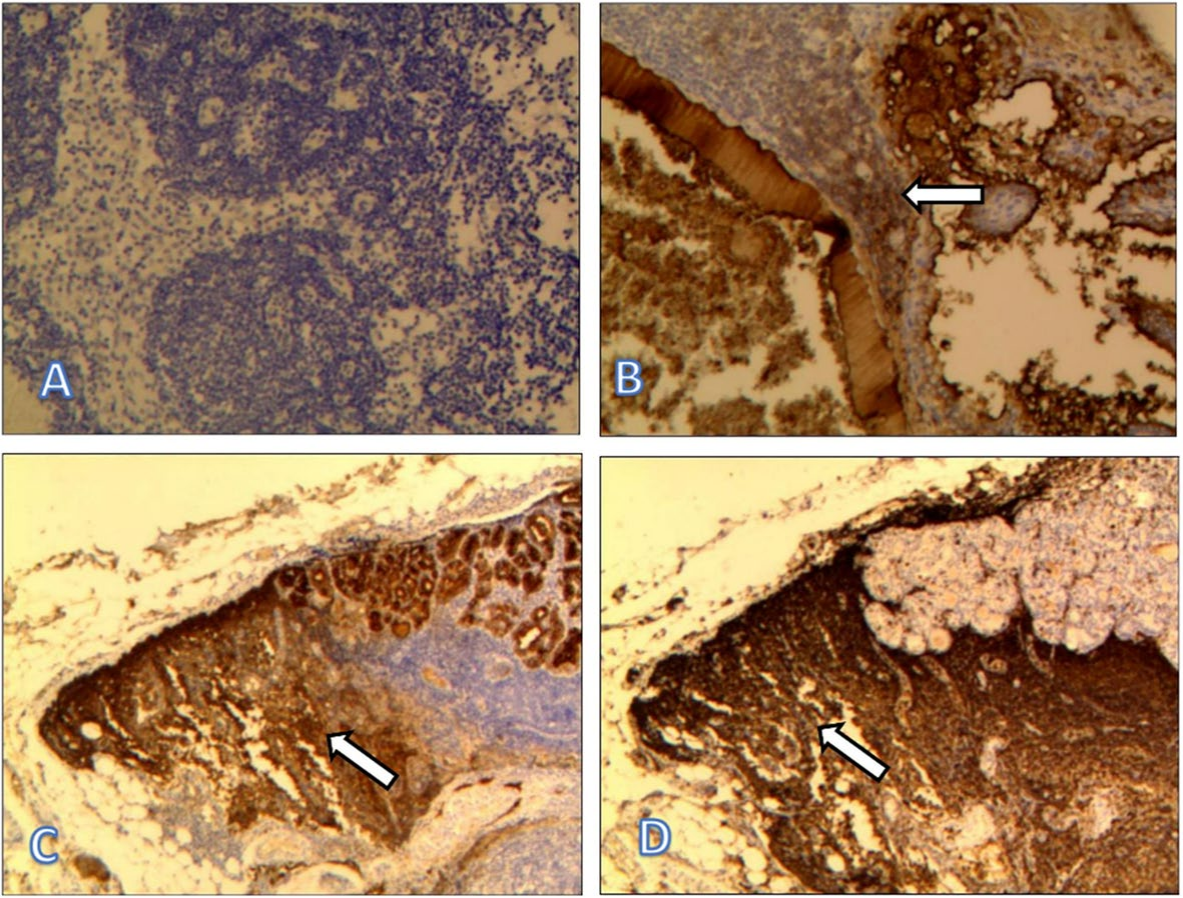
Immunohistochemical staining of lymph nodes with thyroglobulin (**A**/**B**/**C**) and leukocyte common antigen (**D**). **A**, **B**, **C** Tg staining in cytoplasmic regions. **D** LCA staining in lymphocytes adjacent to cancer cells

In this study, some LNs were labeled with immunohistochemical Tg. Because the Tg staining is cytoplasmic, the results were not clear (as shown in A). The probability of false results is high with this approach. Therefore, not all LNs were stained and counted. However, in some lymphoid tissues, Tg markers were found near the cancer nests (as shown in B and C). LCA is a broad-spectrum marker for lymphocytes. Tg and LCA staining were performed on two adjacent sides of the same LN (as shown in C and D) to rule out false-positives. LCA and Tg staining of lymphocytes is shown in D and C, respectively. Once again, the presence of Tg in normal lymphocytes adjacent to cancer cells was confirmed. It also showed that even if fine needle aspiration did not puncture the tumor cells, there was a greater probability of puncture showing Tg (Figs. 3 and 4).

### Evaluation of the cutoff value of FNA-Tg by ROC curve

**Fig. 5:**
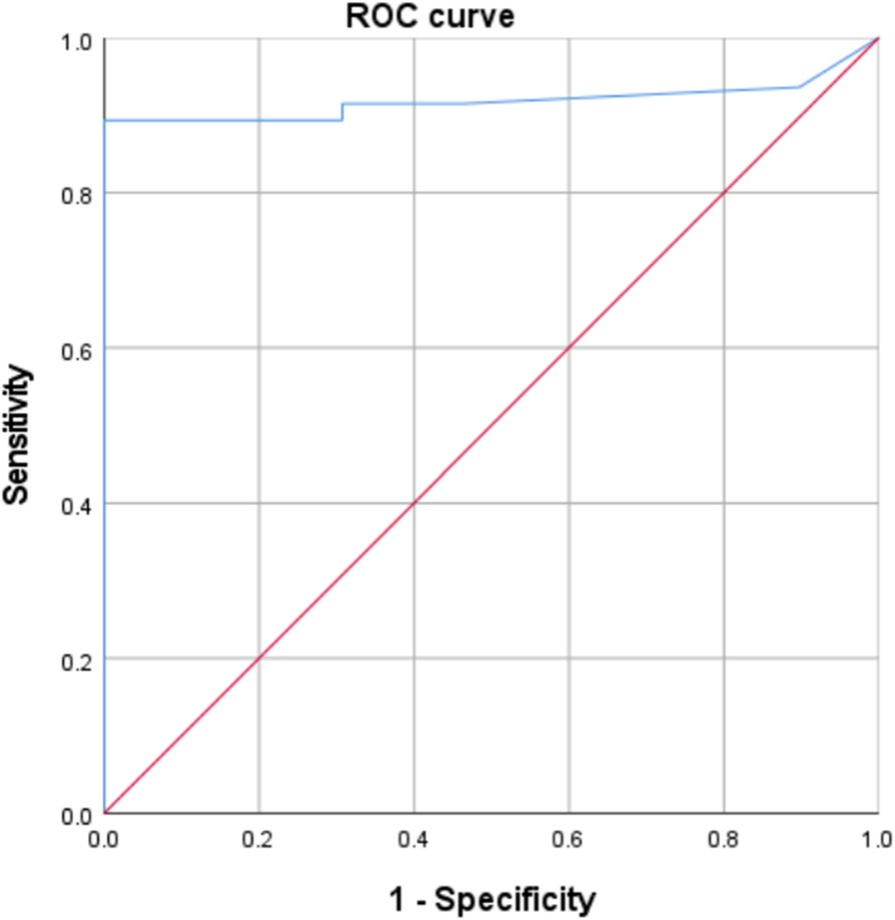
ROC curve of FNA-Tg levels for evaluating lymph node metastasis

The range of FNA-Tg detected in our hospital was from above 300 ng/mL to less than 0.11 ng/mL. ROC curves were drawn according to the accuracy of LN metastasis assessment. The area under the curve was 0.918. The cutoff value was 3.2 ng/mL (Fig. 5).

### Comparing the effectiveness of US, FNAC, FNA-Tg, and FNAC + FNA-Tg in diagnosing LN metastasis in PTC patients

The diagnostic performance of US is shown in Table 2. Increased blood flow had the highest sensitivity; cystic degeneration had the highest specificity. The US results were based on a comprehensive ultrasound interpretation in evaluating LN metastasis. US had a 92.42 sensitivity, but the specificity was not satisfactory (55.1). FNA-Tg had a higher sensitivity (92.42 vs. 89.39), specificity (100 vs. 93.88), and accuracy (92.42 vs. 83.27) than FNAC. However, the sensitivity of FNAC + FNA-Tg increased further, while the specificity and accuracy decreased slightly.

**Table 2:** Diagnostic performance of the different modalities used in the correlation with FNAC results

Test	Sensitivity (%)	Specificity (%)	NPV (%)	PPV (%)	Accuracy (%)
Aspect ratio > 1	46.97	75.51	51.39	72.09	22.48
Increased blood flow signals	89.39	61.22	81.08	75.64	50.61
Cystic degeneration	6.06	100	44.14	100	6.06
Uneven Echo	65.15	57.14	54.90	67.19	22.29
The lymphatic hilum blurred or disappeared	31.82	91.84	50.00	84.00	23.66
Cortical thickening	63.64	83.67	63.08	84	47.31
US	92.42	55.1	84.38	73.49	47.52
FNAC	89.39	93.88	86.79	95.16	83.27
FNA-Tg	92.42	100	90.2	100	92.42
FNAC + FNA-Tg	93.94	93.88	92	95.38	87.82

## Discussion

Surgery is the primary treatment for PTC, a differentiated thyroid cancer. However, some patients present with recurrent metastasis, especially in cervical LNs, and recurrent metastasis is an important factor affecting the prognosis and quality of life. Xu-hang Zhu et al. reported that approximately 30–80% of patients had LN metastasis at initial diagnosis [7]. Therefore, adequate preoperative evaluation of cervical lymph nodes directly impacts the establishment of the operation program, the effect of surgery, and the quality of life.

US can provide a multidimensional comprehensive evaluation of suspicious lymph nodes [8]. The more features that are matched, the greater the US accuracy. PTC patients in the early stage of LN metastasis mainly show increased blood flow and aspect ratio, blurred or absent lymphatic hilum, and cortical thickening. As the disease progresses, LNs may appear ill-defined and fused. Table 3 and Fig. 2 show that an aspect ratio > 1, increased blood flow signals, uneven echo, blurred or absent lymphatic hilum, and cortical thickening afforded a better indication of LN metastasis. LN cystic degeneration appeared in four cases among the LN + , and the specificity was the highest. Metastatic, cervical infectious, and hypermetabolic diseases all lead to abundant lymphadenopathy and blood supply that affect the ultrasound blood supply evaluation and make it difficult to differentiate. This leads to high sensitivity, low specificity, and low accuracy. This does not meet the needs of surgeons. However, US offers irreplaceable advantages for early screening of LNs with a dynamic multiangle noninvasive evaluation.

**Table 3:** Different ultrasonic signs of lymph node metastasis

Different ultrasonic signs	LN ( +) *n* = 66	LN (-) *n* = 49		Value
Aspect ratio > 1	31(46.97)	12(24.49)	6.071	0.014
Increased blood flow	59(89.39)	19(34.69)	31.208	< 0.001
Cystic degeneration	4(6.06)	0(0.00)	3.077	0.079
Uneven Echo	43(65.15)	21(42.86)	5.663	0.017
The lymphatic hilum blurred or disappeared	21(31.82)	4(8.16)	9.249	0.002
Cortical thickening	42(63.64)	8(16.33)	25.613	< 0.001

Cervical LNs are numerous, their size is changeable, and the cervical structure is abundant, containing many important blood vessels and nerves. FNAC is a real-time and dynamic monitoring puncture under ultrasound guidance, providing cytological evidence of high safety and accuracy (As shown in Fig. 3). Furthermore, it has a high diagnostic yield (94.4%) and cellular sample rate (82.2%) [8]. Joshua Vic Chen reported that as the technique matured, the success rate of puncture was similar for LNs of different sizes [8].

The results (Table 2) showed that the sensitivity, specificity, and accuracy of FNAC were significantly higher than those of US, with an accuracy of 83.27%. However, it could be affected by many factors, such as the puncture technique, smear, reading, and so on. For example, in the early stage, tumor cells invade only part of the lymph nodes. Consequently, rich blood flow may affect the puncture process, resulting in false-negative results [9, 10]. Although the accuracy of FNAC is improved, the false-negative rate is not satisfactory.

FNA-Tg does not depend on cell morphology. It is based on FNAC to detect residual Tg in the puncture-needle tract. The LNs in PTC metastasis secrete Tg. An abnormal elevation of Tg is strong evidence of thyroid-derived tumor cells in LN tissue. Studies suggest that this method has a high accuracy rate for evaluating LNs [11, 12].

This is because FNA-Tg can compensate for the deficiency of cytology, especially for PTC metastatic LNs with cystic degeneration. The results (Table 1) demonstrate that s-Tg and FNA-Tg were significantly higher in LN metastasis. FNA-Tg was nearly 300 times higher, and the difference was more obvious for the LN + group. Table 4 further confirmed that FNA-Tg levels were not affected by the number of punctures in most cases. However, the increase in FNAC puncture times can cause bleeding or puncture deviation from the tumor, which can lead to false-negative results.

**Table 4:** FNA-Tg values were measured after lymph nodes were punctured at different times

LN	Puncture 5 times (ng/ml)	Puncture 10 times (ng/ml)
Tg-II (L)	300	60.3
Tg-III (L)	35.5	300
Tg-IV (L)	0.2	0.24
Tg-V (L)	9.48	7.6

However, the FNA-Tg cutoff value remains controversial. Moon et al. [13] recommended a 1.0 ng/mL cutoff value, while Sohn et al. [14] found that 5 ng/mL yielded the best diagnostic performance, and Park [15] suggested 32 ng/mL. Our ROC curve (Fig. 5) using FNA-Tg of LN pathology results yielded a cutoff value of 3.2 ng/mL.

We used 3.2 ng/mL as a criterion to evaluate FNA-Tg. The results showed that the sensitivity and specificity of FNA-Tg improved, with an accuracy of 92.42%. This conclusion is consistent with those of Yuxuan Wang et al. [5] However, other studies showed that the level of FNA-Tg bore no correlation with the number of tumor cells but might be related to the level of hormone secretion by tumor cells. Therefore, if the eluate is abnormally high, it may indicate PTC LN metastasis, but it does not mean that the higher the Tg value, the greater the possibility (Table 4).

We performed Tg and LCA immunohistochemical staining on some LNs to further demonstrate the accuracy of FNA-Tg. The result (Fig. 4) showed that Tg might be present near the cancer nest in some lymph nodes. We performed lymphocyte LCA labeling on adjacent sections that suggested the presence of Tg in normal lymphocytes to rule out false-positives and demonstrated the presence of elevated Tg in the surrounding tissue. Even though the tumor cells were not punctured, the high level of FNA-Tg was indirect evidence of LN metastasis.

In summary, ultrasonography, used as an early screening method, offers a noninvasive, dynamic, and multidimensional assessment of LN status. It remains the first choice for screening PTC lymph node metastasis. With a cutoff value of 3.2 ng/mL, FNA-Tg has higher accuracy and a lower false-negative rate than various single diagnoses. FNAC combined with FNA-Tg to evaluate LNs has higher accuracy and lower false rate than FNAC, higher sensitivity, and lower accuracy than FNA-Tg. FNA-Tg does not cause additional pain and has higher diagnostic efficacy and clinical value.

### Supplementary Information

Supplementary Material 1.

Supplementary Material 2.

Supplementary Material 3.

Supplementary Material 4.

Supplementary Material 5.

Supplementary Material 6.

## Data Availability

The datasets used or analyzed during the current study are available from the corresponding author on reasonable request.
