# Supplementary figures and images for: The efficacy and assessment value of the level of thyroglobulin wash-out after fine-needle aspiration cytodiagnosis in the evaluation of lymph node metastasis in papillary thyroid carcinoma
Source: World J Surg Oncol. 2024 Jun 5;22:149. doi: 10.1186/s12957-024-03430-5 (PMC11151531; doi:10.1186/s12957-024-03430-5)

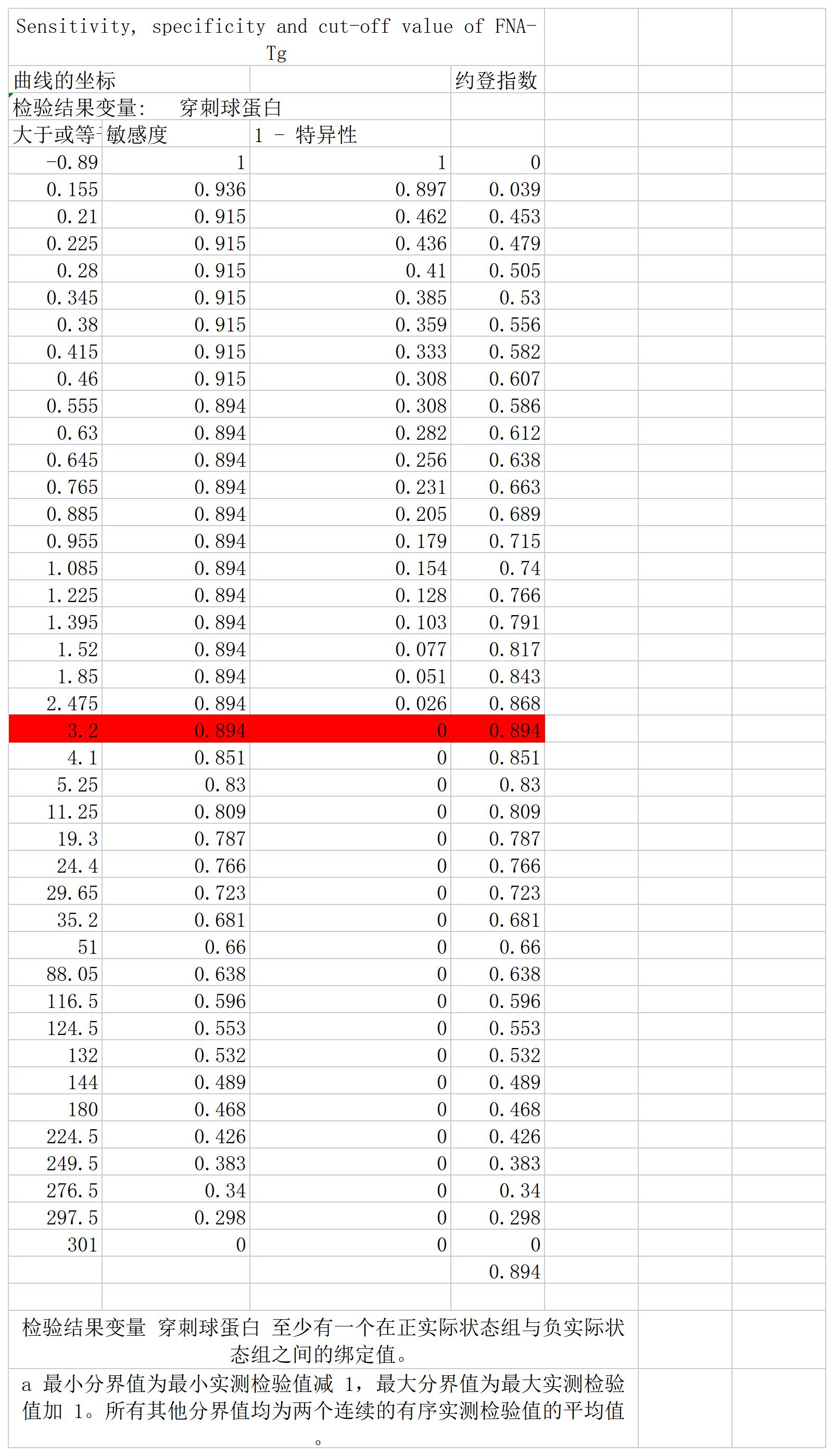

Supplement: Supplementary file 1 — Supplementary Material 1. [file 12957_2024_3430_MOESM1_ESM.jpg]

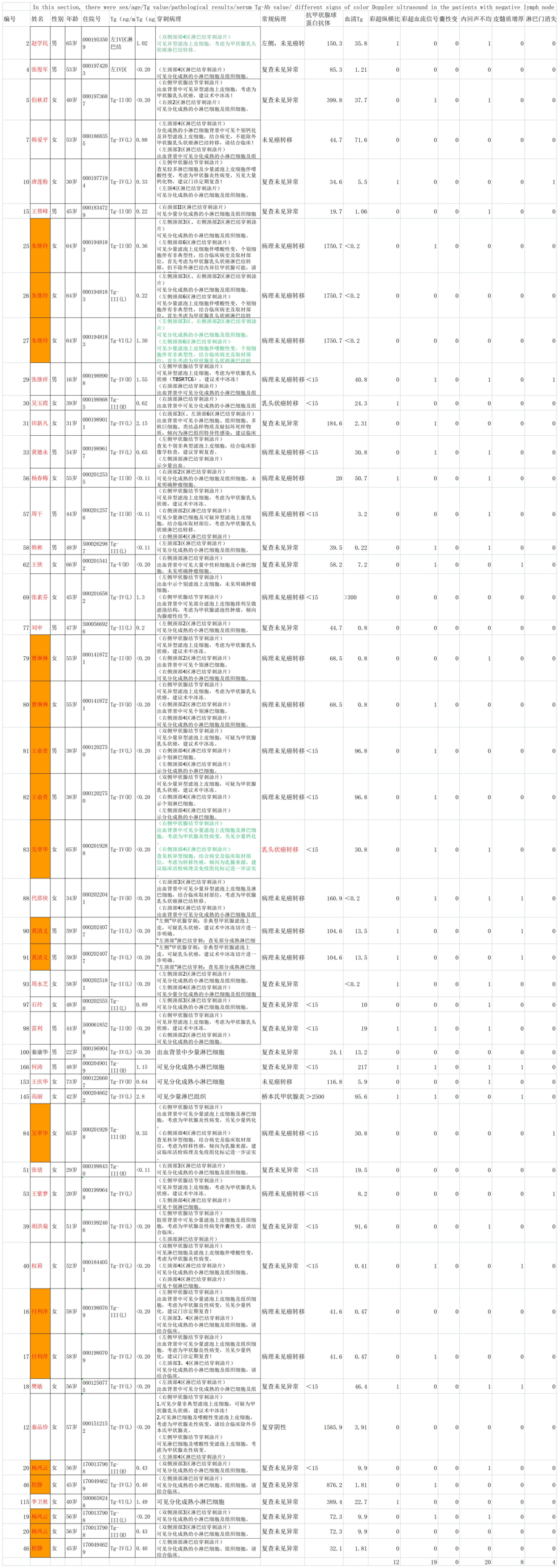

Supplement: Supplementary file 2 — Supplementary Material 2. [file 12957_2024_3430_MOESM2_ESM.jpg]

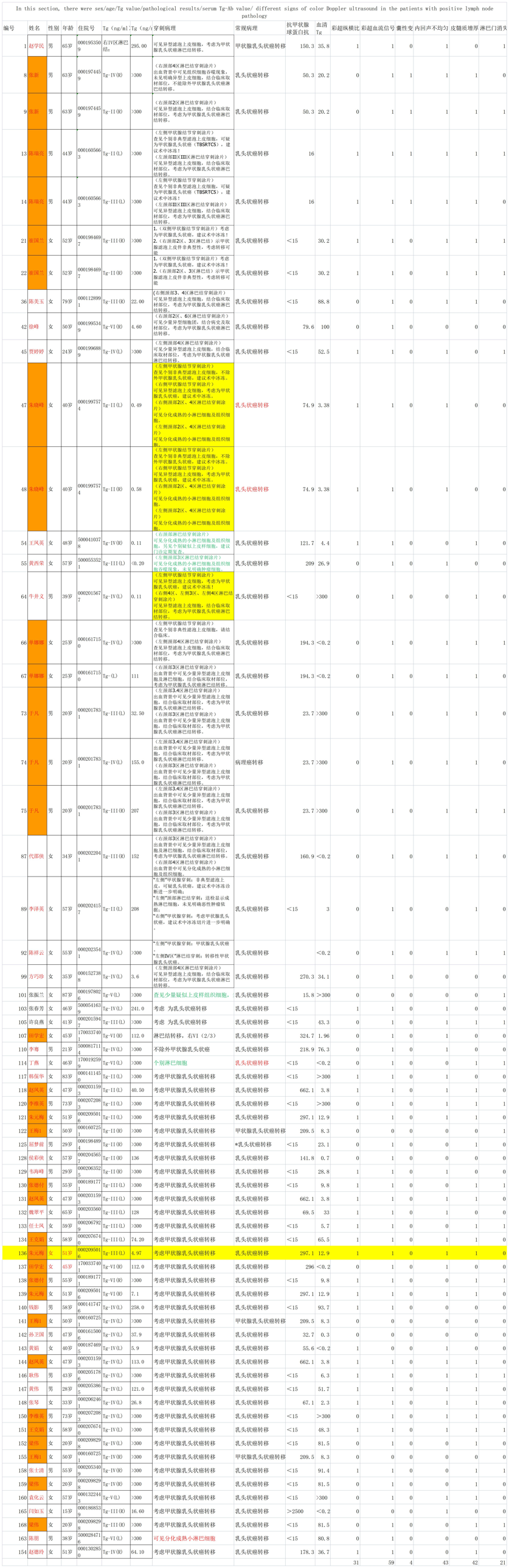

Supplement: Supplementary file 3 — Supplementary Material 3. [file 12957_2024_3430_MOESM3_ESM.jpg]
